# Clinical Manifestation and Risk Factors of Tuberculosis Infection in Malaysia: Case Study of a Community Clinic

**DOI:** 10.5539/gjhs.v7n4p110

**Published:** 2014-12-31

**Authors:** Rohan Shanmuganathan, Indra Devi Shanmuganathan

**Affiliations:** 1 Department of Internal Medicine, Hospital Sungei Buloh, Selangor, Malaysia; 2 Graduate School of Management, Multimedia University, Cyberjaya, Malaysia

**Keywords:** Tuberculosis, clinical manifestation, risk factors, Malaysia

## Abstract

**Introduction:**

The main aim of this study was to describe the clinical manifestation of tuberculosis infection cases in Malaysia and to determine the individual risk factors for their occurrence.

**Methodology:**

The study adopted a quantitative research approach with use of descriptive statistical approach. The study setting was a community clinic which treats walk in patients who are mainly living and working in the surrounding areas. The study was conducted for a period of one year. All tuberculosis patients who sought treatment in the clinic during the time were included in this study. The total number of cases was 40. Data was collected from the medical records of the tuberculosis patients. The risk factors selected for investigation were demographic characteristics of age and sex, personal habits such as smoking, drug use and alcohol and presence of diseases such as human immunodeficiency virus positive (HIV+), diabetes mellitus, cancer, cyanotic heart disease, renal failure and steroid use.

**Results:**

Patients in the age group ranging from 41 to 50 years had the highest incidence of the infection. Smoking appears to be the most important risk factor for contracting followed by drug abuse, HIV+ infection and diabetes mellitus.

**Conclusions:**

People with diseases such as diabetes mellitus and HIV that are high risk factors for TB should be screened for TB so that early detection and intervention is possible. Educational programs should be carried out to create awareness among the at risk groups.

## 1. Introduction

### 1.1 Incidence of Tuberculosis Cases

Tuberculosis (TB) cases are on the rise more so in developing countries. According to Davies (2003) and Dye (2006), TB is foremost among diseases that cause mortality worldwide. The statistics indicate that 8.6 million people were infected with this disease in 2012 and the death due to the disease was about 8.6 million of which about a quarter were people who had been diagnosed positive for HIV. Many of these deaths could have been averted through treatment.

Among the cases worldwide, South East Asia tops the list with 29% followed by Africa (27%), and Western Pacific regions (19%). The two largest populated nations of the world also recorded high incidence of TB cases with India accounting for 26% and China registering 12%. The incidence of TB cases is significantly lower in developed countries (WHO, 2013).

Coinfection of TB with HIV is on the rise. According to the Global TB Report 2013, among those who were infected with TB in 2012, HIV positive persons constituted about 13% off the total number. Further cause for worry is the emergence of multi-drug resistant TB cases worldwide estimated to be 450,000 for the same year with about 170,000 deaths being attributed to it.

Although the incidence of TB infection and death due to the disease is higher among men than women, it is among the top three causes of death for women. Of the 8.6 million new cases of TB globally in 2012, a third of them were women. Coinfection with HIV was responsible for the death of 160,000 of the 450,000 women who died of TB in 2012. Children under 15 years old represented 6% of TB cases worldwide and 8% of the mortality among HIV negative TB cases were children (WHO, 2013).

According to Frieden, Sterling, Munsiff, Watt and Dye (2006), TB can have detrimental effect on the economic situations of individuals and nations as most active cases of TB occur in persons between the ages of 19 and 49. It is during this phase of their lives that people are actively involved in their career and contribute towards a nation’s Gross Domestic Product (GDP).

### 1.2 Cause of TB and Its Transmission

TB is an infectious disease. It is caused by the bacteria Mycobacterium tuberculosis (Kumar, Abbas, Fausto, & Mitchell, 2007). The *Mycobacterium tuberculosis* possesses a distinct cell wall which is necessary for the bacteria’s survival because it contains a fatty acid called mycolic acid which provides a strong lipid barrier (Cantrell, Leavell, Marjanovic, Iavarone, Leary, & Riley, 2013).

It is the physiological characteristics of this barrier that is responsible for problems in treating the disease because it provides the bacteria with resistance to antibiotics and the normal immunological mechanisms possessed by the host. The components of the cell wall both in terms of composition and quantity affect the bacteria’s aggressiveness and escalation rate (Lee, Li, Chatterjee, & Lee, 2005). The peptidoglycan polymer external to the bacterial cell membrane which gives the cell wall its rigidity adds to its impermeability. The survival of the mycobacteria within macrophages is facilitated by another cell wall constituent called lipoarabinomannan. This antigen is immunogenic and exists on the outside of the organism (Lee et al., 2005; Joe, Bai, Nacario, & Lowary, 2007).

TB mostly attacks the lung. This type of infection is called pulmonary TB. TB can also infect organs other than the lung such as the lymphatics, pleura, bones/joints, or meninges. In the latter cases it is referred to as extrapulmonary TB. TB is spread by droplets released into the air by pulmonary or laryngeal TB patients when they cough or sneeze. These droplets are durable staying in the air several hours after their expulsion (Lee et al., 2005). According to the American Thoracic Society and Centers for Disease Control and Prevention (2000) many variables can influence the spread of the disease. Among these are the bacilli’s number in the droplets, their aggressiveness, and the extent of their exposure to UV light and the availability of ventilation. Not all people who are infected with the bacteria go on to develop the disease. The probability of developing the disease is higher among persons exposed to risk factors. Therefore initiatives to control or prevent the disease would require addressing the risk factors.

### 1.3 Pathophysiology

When an individual inhales, the droplets containing the bacteria are dispersed throughout the person’s airways but mainly on its upper part where mucus producing cells are present. Most of the bacilli are caught here. When this happens, the cilia present on the cells start pushing the mucus and the trapped bacilli upwards in an attempt to remove them from the body (Frieden, Sterling, Munsiff, Watt, & Dye, 2003). This action is the first defence mechanism by the body to prevent infection by the bacteria (Jensen, Lambert, Iademarco, & Ridzon, 2005).

Those that escape this system, end up in the alveoli. The alveoli macrophages are immune effectors cells that are present in large numbers in alveolar spaces (Korf, Pynaert, Tournoy Boonefaes, Van Oosterhout, Ginneberge, Haegeman et al., 2006.). The macrophages are the primary host cells for the *Mybacterium tuberculosis*. These macrophages are phagocytes and are part of the body’s natural immune system whose function is to eliminate invading pathogens and prevent onset of diseases (Van Crevel, Ottenhoff, & Van der Meer, 2002). The macrophages surround and engulf the mycobacteria in the droplets that end up in the alveoli (Frieden et al., 2003).

Lipoarabinomannan (LAM) is one of the key virulence factors for the bacteria. and plays a significant role in its phagocytosis (Li, Petrofsky, & Bermudez, 2002). This compound in the cell wall of the mycobacteria interacts with the C3 proteins in the cell membrane of the host macrophage and enables recognition of the mycobacteria by the macrophage facilitating its engulfment by the latter. This results in phagocytosis of the mycobacteria.

A series of events result as a consequence of continuing phagocytosis of the mycobacteria by the macrophages. The infection could be controlled with the TB present in the host in a latent state or it could also lead to manifestation of the disease in its active form which is referred to as primary progressive TB (Frieden et al., 2003). The quality of the defenses possessed by the host and the strength of host defenses against the virulence of the mycobacteria will determine which of these two outcomes happen (Goyot-Revol, Innes, Hackforth, Hinks, & Lalvani, 2006).

Post ingestion by macrophages, the mycobacteria will continue to increase in numbers (Frieden et al., 2003) through cell divisions taking place at regular intervals (Porth, 2002). Mycobacteria infecting macrophages live in cytoplasmic vesicles that resist fusion with lysosomes and consequent destruction of the bacteria by macrophage bacteriocidal activity. Irrespective of the stage of development of the infection, the first initiative by the macrophages is to produce proteolytic enzymes and cytokines to try to weaken the mycobacteria. The cytokines attract T cells to the spot. The macrophages respond by presenting mycrobacterial antigens to the T cells. It is the start of the development of cell-mediated immunity van Crevel, Ottenhoff, Jos and van Meer, (2002).

The progress of the disease depends essentially on the patient’s immune system. The differences in stages of the TB as described by van Crevel, Ottenhoff, Jos and van Meer (2002) are presented in Table 1. These stages include latency, primary disease, primary progressive disease, and extrapulmonary disease. The manifestation of the disease is different in each stage.

**Table 1:** Differences in the stages of tuberculosis

Early infection	Early primary progressive (active)	Late primary progressive (active)	Latent
-The host’s immune system combats the infection. -Progression of the disease takes place in the absence of signs or symptoms. -Patients could develop fever, paratracheal lymphadenopathy, or dyspnea. -The Infection may not progress to active stage but remain in a subclinical condition.	-The initial infection is not controlled by the host’s Immune system -The tissues become inflamed -Patient exhibits nonspecific signs or symptoms for example fever, loss of weight and fatigue. -Cough that develops in this stage is nonproductive. -Result of chest radiograph is normal and smear tests for sputum is negative for mycobacteria making diagnosis difficult.	-Cough in this stage is productive. -With the progress of the disease, signs and symptoms increase. -Patient suffers more weight loss, rales and anemia. -Result of chest radiograph is normal. -Cultures of sputum are used for diagnosis of the disease.	-The Mycobacteria continues to inhabit the host’s body. -Absence of signs or symptoms. -Patient does not feel sick. -Patient is at risk of reactivation of the disease. -Chest radiograph shows calcified granulomatous lesions which have become fibrotic. -There is likelihood of reappearance of the infection when risk factors present in the hosts cause immunosuppression.

### 1.4 Background of the Problem

There has been a steady increase in yearly notification of new cases of TB in Malaysia over the past 20 years. The absolute number of new cases has been increasing from about 15,000 new cases in 2002 up to 16,665 in 2006 (Ministry of Health, Malaysia, 2008). As of 2010, the TB case detection rate (%, all forms) in Malaysia was 80.00, its highest value over the past 20 years. TB case detection rate (all forms) is the percentage of newly notified TB cases (including relapses) to estimated incident cases (case detection, all forms) (WHO, 2010). TB is the second highest contagious disease in Malaysia with 22,710 cases in 2012. It is the highest cause of death among contagious diseases. In 2013 it claimed 1,597 lives as compared to 1,414 cases in 2012 (Subramaniam, 2014).

While it is important that appropriate and effective measures are used to treat those infected with TB, it is just as important to take actions to prevent the occurrence of the disease. One way of preventing the occurrence of the disease is to control its transmission from infectious TB cases. For an individual who has been infected with TB after exposure to infectious cases, the development of TB takes a two stage process. For those with strong immune system, the infection is contained. About 10–15% of those infected go on to develop active disease at some stage of their lives (Maher, 2009).

To prevent infection and manifestation of the disease, it is important understand the factors that contributes to both these states. The first step in control of TB is to understand the risk of infection after an individual has been exposed to infectious cases. According to Narasimhan, Wood, Raina, MacIntyre, and Mathai (2013) this is governed by a combination of factors that include the acuteness of the infectious source case, the physical closeness of the individual to the source case and the extent to which the individual possesses the social and behavioural risk factors such as smoking, alcohol, air pollution and overcrowded situations. In these conditions the transmission of the disease is high. Delay in diagnosis could also prolong the individual’s exposure to the infectious patients and could therefore be another contributory factor.

The second step in control of the disease requires an understanding of the factors that accelerate the progression of infection to disease. All conditions that alter the immune response of the host increase the risk of progression to the disease. Among these are HIV, Diabetes, alcohol, malnutrition, tobacco smoke, and indoor air pollution. The prevalence of these factors vary from country to country accounting for the differential impact of these factors on the larger section of the population and progression from infection to TB disease. Therefore to prevent and control TB in Malaysia requires an understanding of the risk factors that are specific to the local context. This study aims to address this problem.

## 2. Methodology

### 2.1 Research Design

**Figure 1:**
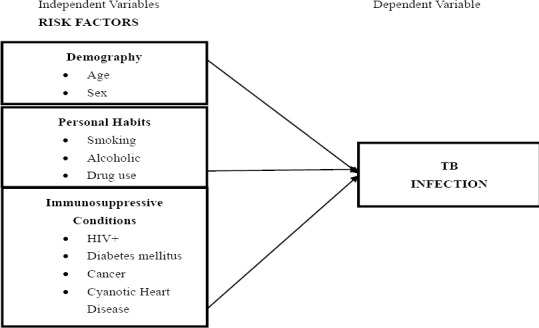
Research Framework

The study adopts a quantitative approach and a descriptive research design. The research framework is presented in Figure 1.

### 2.2 Population and Sample

The study setting is a community clinic which treats walk in patients who are mainly living and working in the surrounding areas. The study was conducted from 1 January 2012 to 31 December 2012. All TB patients who sought treatment in the clinic during the time were included in this study. It is therefore a census study. The total number of patients who fit this description was 40. Since this study was carried out in one community clinic and comprised of outpatients, the findings are only generalizable to that clinic. However if the TB patients in this study are representative of microcosm of the TB patients in Malaysia, the finding of the study could be an indicator of the characteristics of TB patients in rest of Malaysia. Patients of the clinic are mainly those living and working in the surrounding areas.

### 2.3 Data Collection

The sources of data are the TB patients’ medical records. The data was obtained retrospectively by reference to these cards. The medical records are in the form of cards and the information contained in them include demography of the patients (sex, age, nationality, race, marital status), personal habits (smoke, drink alcohol, take drugs), whether they suffer from other diseases (HIV/AIDS, diabetes mellitus, cancer, diseases of the liver), type of case, registration category and treatment outcomes. To ensure compliance with research ethics, the identity of the patients was not revealed and the data are analyzed collectively.

### 2.4 Data Analysis

Descriptive statistics in the form of frequency and percentages was used to analyze the data and answer the research questions and objectives. Statistical tests in the form of chi square are required to determine whether there is significant association between risk factors and TB. However this test could not be conducted because the sample size was too small resulting in some cells being empty.

## 3. Results

### 3.1 Demographic Profile of the Respondents

The profile of the respondents in terms of demography is presented in Table 2.

**Table 2:** Demographic Profile of Respondents

Variables	Frequency	Percentage
*Sex*		
Male	31	77.5
Female	9	22.5

*Age Group (Years)*		
20 – 30	8	20
31-40	11	27.5
41-50	12	30
51-60	8	20
>60	2	2.5

*Nationality*		
Malaysian	32	80.0
Non-Malaysia	7	20.0

Race		
Malays	22	45
Chinese	5	12.5
Indian	5	12.5
Indonesian	7	17.5
Cambodian	1	2.5

*Marital Status*		
Single	16	40
Married	24	60

*Occupation*		
Employed	14	35
Unemployed	26	65

There were more male patients (77.5%) compared to female patients (22.5%). The distribution of the patients by age is 20-30 years (20%), 31-40 years (27.5%), 41-50 years (30%), 51-60 years (20%) and those above 60 years constituted the remaining 2.5%. Majority of the patients were Malaysian (70%) with Malays forming the largest proportion (45%). Chinese and Indians with 12.5% each made up the remaining 25%. Indonesian was the majority (25%) among the non-Malaysian. The remaining 2.5% were Cambodians. With regards to marital status 60% were married while the remaining 40% were single. A large proportion (65%) was unemployed.

### 3.2 Category of Cases

Table 3 describes the patients according to category of cases. Majority of them were new cases (87.5%), 3 of them were treatment after interruption, there was 1 case each (2.5%) for relapse and treatment failure.

**Table 3:** Category of Cases

Category of cases	Frequency	Percentage
New Case	35	87.5
Relapse	1	2.5
Treatment Failure	1	2.5
Treatment after Interruption	3	7.5

### 3.3 Clinical Manifestation of the Disease

Table 4 presents the clinical manifestation of the disease among the cases that were studied. Most of the cases were pulmonary TB (90%) with only 10% being extra pulmonary TB.

**Table 4:** Clinical Manifestation of the Disease

Variables	Frequency	Percentage
*Diagnosis*		
Pulmonary TB	36	90
Extra Pulmonary TB	4	10

*Symptoms and Signs*		
Prolonged cough	5	12.5
Prolonged cough + sputum	4	10.0
Prolonged cough + bloody sputum	1	2.5
Prolonged cough + bloody sputum + weight loss	5	12.5
Prolonged cough + weight loss Sputum + weight loss but no cough	11	27.5
Prolonged cough + bloody sputum +weight loss + fever + night sweat	3	7.5
Prolonged cough + bloody sputum + fever + no appetite	2	5.0
Prolonged cough + sputum + weight loss + fever + sweat at night	6	15.0
Prolonged cough + bloody sputum + weight loss + fever + no appetite
	2	5.0
	1	2.5

*Results of investigation using sputum direct smear for acid fast bacilli (AFB)*		
Sputum with one smear positive	20	50
Sputum with two smear positive	4	10
Sputum with three sputum smear positive	13	32.5
Sputum with smear negative	3	7.5

The TB patients displayed several different symptoms. These included prolonged cough (12.5%), prolonged cough with sputum (10%), prolonged cough with bloody sputum (2.5%), prolonged cough with bloody sputum and weight loss (12.5%), prolonged cough with weight loss (27.5%), sputum with weight loss but no cough (7.5), prolonged cough with bloody sputum, weight loss, fever and night sweat (5%), prolonged cough with bloody sputum, fever and no appetite (15%), prolonged cough with sputum, weight loss, fever and sweat at night (5%) and prolonged cough with bloody sputum, weight loss, fever and no appetite (2.5%).

### 3.4 Result of Sputum Direct Smear Test for Acid Fast Bacilli (AFB)

Table 5 presents the result of testing for TB using the sputum direct smear for acid fast bacilli (AFB) found positive results for 92.5% of the cases while the remaining 7.5% tested negative.

**Table 5:** Results of investigation using sputum direct smear for acid fast bacilli (AFB)

Results	Frequency	Percentage
Sputum with one smear positive	20	50
Sputum with two smear positive	4	10
Sputum with three sputum smear positive	13	32.5
Sputum with smear negative	3	7.5

### 3.5 Presence of Risk Factors

The distribution of the patients according to whether they possess selected risk factors is presented in Table 6.

**Table 6:** Distribution of cases by presence of risk factors

Risk Factors	Frequency	Percentage
Drugs User		
Yes	13	32.5
No	27	67.5

Smoke		
Yes	19	47.5
No	21	52.5

Drink Alcohol		
Yes	2	5
No	38	95

HIV Positive		
Yes	5	12.5
No	35	87.5

Diabetic mellitus		
Yes	5	12.5
No	35	87.5

Cancer		
Yes	0	0
No	40	100

Cyanotic Heart Disease		
Yes	1	2.5
No	39	98.5

Renal failure		
Yes	1	2.5
No	39	98.5

Steroid treatment		
Yes	1	2.5
No	39	98.5

Distribution by personal habits showed that a nearly a third of them (32.5%) were drug users; nearly half of them smoked (47.5%) and (5%) drank alcohol. The results show 12.5% of the patients had HIV, 12.5% were diabetic, 2.5% each had cyanotic heart disease, renal failure and steroid treatment. None of the cases had cancer. Risk factors according to rank would be (1) smoking, (2) drug use, (3) HIV and diabetes mellitus (5) alcoholic (6) heart disease, renal failure and steroid use. Cancer does not appear to be a risk factor.

## 4. Discussion

The main aim of this study was to determine whether there was association between selected risk factors and TB among Malaysians infected with the disease. The risk factors selected for investigation were demographic characteristics of age and sex, personal habits such as smoking, drug use and alcohol and presence of diseases such as HIV and diabetes mellitus, cancer, cyanotic heart disease, renal failure and steroid use. Other risk factors which have been identified in literature such as malnutrition, poverty, living in overcrowded dwellings and exposure to immigrants from countries with high incidence of TB could not be studied as these data were either incomplete or non-existent in the patients’ medical cards.

Majority of the TB cases in this study were male. It does indicate that sex could be a risk factor for TB infection. This is similar to the research results of Neyrolles et al. (2009). However, the reason for fewer numbers of women could be due to lower number of notification. Women have been found to not to reveal their infection due to stigma attached to the disease. About a quarter of the cases were over the age of 50. However the age group with the highest percentage was between 41 and 50 years of age. Since there were no cases in the infant and very young age category, again it was difficult to make and conclusions about babies and young children being more susceptible to TB because their immunity would have developed fully as was stated by Marais et al. (2004). Smoking appears to be the most important risk factor for contracting TB among Malaysians. This is consistent with the research findings of Bates et al. (2007) and Lin et al. (2007). Impaired clearance of mucosal secretion and reduced phagocytic activities of the alveolar macrophages are some of the reasons for smokers being at high risk for TB infection. Drug use registered second highest frequency among the TB patients. Markowitz (1993), Friedman et al. (1996) and Kaizer et al. (2000) all had similar results. Impaired immune system both natural and cell mediated have been cited as some of the reasons for drug use being a risk factor especial in latent cases. One in eight of the TB patients were HIV suggesting that the latter infection could be a risk factor. Corbett et al. (2003) found HIV infection in patients reduced their immunity making them highly susceptible to TB infection. Similar results were found by Lawn et al. (2006) and De Riemer et al. (2007). Diabetes mellitus also appears to be a risk factor for TB infection among Malaysians. This finding is consistent with that of Alisjahbana et al. (2007) and Baker et al. (2007). The presence of diabetes impairs the natural and adaptive immune responses to TB. There was 1 case each for renal failure, heart disease and steroid use and none for cancer. The little or no presence of this disease among the TB cases that was studied could be due to chance.

## 5. Conclusion

People with diseases such as diabetes mellitus and HIV that are high risk factors for TB should be screened for TB so that early detection and intervention is possible. Educational programmes should be carried out to create awareness in people regarding the dangers of smoking; alcoholism and drug use especially their susceptibility to TB. There should be aggressive vaccination campaign specifically for the very old and the very young and among men so that their vulnerability to TB is reduced.

It is noted that this study has several limitations. Since data was collected from the TB patients’ medical records, only information collected in the cards could be used as variables in the study. This means that other risk factors such as malnutrition (indicated by body mass), living conditions, their social environment, age variations were not available to be used as variables in the study. The study was conducted in one community clinic with walk-in TB patients; as a result it is not generalizable to a larger population. In order to address these short comings, further research should be carried out with a larger population and covering bigger geographical area. A research instrument specifically designed for the research consisting of both structured and unstructured questions should be used to collect data.
